# MicroRNA-29c functions as a tumor suppressor by targeting VEGFA in lung adenocarcinoma

**DOI:** 10.1186/s12943-017-0620-0

**Published:** 2017-02-28

**Authors:** Lipin Liu, Nan Bi, Lihong Wu, Xiao Ding, Yu Men, Wei Zhou, Lin Li, Weimin Zhang, Susheng Shi, Yongmei Song, Luhua Wang

**Affiliations:** 1 Department of Radiation Oncology, National Cancer Center/Cancer Hospital, Chinese Academy of Medical Sciences and Peking Union Medical College, Beijing, 100021 China; 2 Cancer Center, Shandong Provincial Hospital Affiliated to Shandong University, Jinan, 250021 China; 3 The State Key Laboratory of Molecular Oncology, National Cancer Center/Cancer Hospital, Chinese Academy of Medical Sciences and Peking Union Medical College, Beijing, 100021 China; 4 Department of Pathology, National Cancer Center/Cancer Hospital, Chinese Academy of Medical Sciences and Peking Union Medical College, Beijing, 100021 China

**Keywords:** Lung adenocarcinoma, MiR-29c, VEGFA

## Abstract

**Background:**

Lung adenocarcinoma (LAD) is considered to be a highly aggressive disease with heterogeneous prognosis and the molecular mechanisms underlying tumor progression remain elusive. Growing evidence demonstrates that the dysregulation of microRNAs (miRNAs) plays an important role in various tumor processes. The aim of this study is to discover prognostic miRNA and investigate its role involved in progression of LAD.

**Methods:**

Prognosis related miRNA was detected by miRNA microarray using formalin-fixed paraffin-embedded (FFPE) specimens from 87 patients with IIIA-N2 LAD. The cell proliferation was evaluated by Cell Titer 96 AQueous One Solution Cell Proliferation Assay (MTS), and the migration/invasion was evaluated by transwell assay. The bioinformatics methods and luciferase reporter assay were applied to detect the relationship between miRNA and its target. The mRNA and protein levels of miRNA target were determined by quantitative real time polymerase chain reaction (qRT-PCR) analysis, western blot and enzyme-linked immunosorbent assay (ELISA). Changes of angiogenesis induced by miRNA was evaluated by human umbilical vein endothelial cell (HUVEC) tube formation assay. Immunohistochemistry (IHC) analysis was performed in FFPE specimens of patients to evaluate the correlation between miR-29c with microvessel density (MVD) and vascular endothelial growth factor A (VEGFA) expression.

**Results:**

MiR-29c expression downregulation was significantly associated with unfavorable prognosis in IIIA-N2 LAD. MiR-29c inhibited cell proliferation, migration and invasion in cell lines. Integrated analysis revealed that VEGFA was a direct target of miR-29c. MiR-29c reduced the capability of tumor cells to promote HUVEC tube formation. The compromised cell proliferation, migration/invasion and angiogenesis induced by miR-29c mimic transfection were reversed by transfection of VEGFA expression plasmid. Furthermore, the correlation of miR-29c with MVD and VEGFA was confirmed in patients’ samples.

**Conclusions:**

MiR-29c acts as a tumor suppressor by targeting VEGFA and may represent a promising prognostic biomarker as well as a potential therapeutic target for LAD.

## Background

Lung cancer is the leading cause of cancer-related deaths worldwide [1]. Non-small-cell lung cancer (NSCLC) accounts for 85% of all lung cancer cases and approximately half of NSCLC are lung adenocarcinoma (LAD) [2]. With high invasiveness and early metastasis, LAD often presents with locally advanced or metastatic disease at prognosis [3]. Furthermore, stage IIIA-N2 LAD presents substantial heterogeneity ranging from resectable microscopic lymph node disease to unresectable, bulky nodal metastases [4], with 5-year overall survival (OS) ranging from 13 to 58.3%[5–7]. The current tumor-node-metastasis staging system is inadequate to explain the large variability in disease outcome for stage IIIA-N2 LAD. Moreover, despite treatment efficacy improvement attributable to the target-specific inhibitors to epidermal growth factor receptor (EGFR) or anaplastic lymphoma kinase (ALK), only 15–30% of LAD harbor sensitizing mutation in EGFR or ALK gene rearrangement [8]. Therefore, discovery of novel precise prognostic biomarkers is warranted and a better understanding of molecular mechanisms underlying tumor progression may facilitate the development of effective therapeutic strategies for LAD.

MicroRNAs (miRNAs) are a group of short, noncoding RNAs that negatively regulate gene expression post-transcriptionally by binding to the 3’-untranslated regions (3’-UTR) of their target gene mRNAs, thereby controling a variety of cellular functions such as proliferation, differentiation and apoptosis [9]. Emerging evidence indicates that aberrant expression of miRNAs is closely associated with tumorigenesis and cancer progression via the regulation of key oncogenes or tumor suppressors [10]. Therefore, miRNA may have great potential as a prognostic indicator and therapeutic target [11]. It is well established that miRNA may perform different functions through distinct pathways in a way that depends on the context or tissue specificity [12]. A number of investigations have demonstrated that aberrant miRNA expression contributes to NSCLC progression, such as miR-1, let-7 and miR-21 [13–15]. However, miRNAs identified in the regulation of LAD progression are limited [16, 17].

In this report, we first performed miRNA microarray to survey expression of 1105 known miRNAs in formalin-fixed paraffin-embedded (FFPE) specimens of 87 patients and discovered that downregulation of miR-29c to be significantly related to poor prognosis of patients with IIIA-N2 LAD. We further validated the tumour suppressive role of miR-29c showing evidence that miR-29c inhibits proliferation, migration and invasion in LAD cell lines. We also confirmed that vascular endothelial growth factor A (VEGFA) is a novel, downstream gene target of miR-29c which played an essential role in angiogenesis in vitro.

## Methods

### Patients and tissue specimens

The FFPE specimens of 87 cases with pathologically confirmed IIIA-N2 LAD and available clinical information as well as follow-up information were obtained at the Cancer Hospital, Chinese Academy of Medical Sciences from 2003 to 2005. All the patients underwent radical surgical resection. Patients who underwent preoperative radiotherapy and chemotherapy were excluded. Written informed consent was obtained by all patients and the study was approved by the Institutional Review Boards of Cancer Hospital, Chinese Academy of Medical Sciences.

### MiRNAs microarray and quantitative real time polymerase chain reaction (qRT-PCR) analysis

Total RNA was extracted using Trizol reagent, and miRNAs from both cultured cells and FFPE specimens were obtained using mirVana miRNA Isolation Kit (Ambion, USA). Affymetrix GeneChip miRNA 2.0 Array including probes for 1105 mature human miRNA sequences was performed by a service provider (CapitalBio, Beijing, China).

The expression level of miR-29c was quantified by the stem-loop qRT-PCR method. The cDNA was prepared from total RNA (2 ug) with the miR-29c stem-loop RT primers or U6 RT primers (RiboBio, China) by using the First-Strand cDNA Synthesis kit (Promega, USA). U6 small nuclear RNA was used as an internal control for miR-29c. For quantification of VEGFA, the RNA was reversely transcribed into cDNA with the First-Strand cDNA Synthesis kit (Promega, USA) using oligo primers. Primer sequences of VEGFA were as follows: forward: 5’-CGCAGCTACTGCCATCCAAT-3’; reverse: 5’-GTGAGGTTTGATCCGCATAATCT-3’. β-actin mRNA was used as an internal control for VEGFA transcript. Then, the qRT-PCR was conducted using Light Cycler DNA Master SYBR Green I Mix (Roche, Switzerland) on an ABI Prizm 7300 Sequence Detection System. The miR-29c and VEGFA mRNA were quantified with the 2^-ΔΔCt^ method and normalized with the level of the internal control, respectively.

### Cell culture and transfection

Human LAD cell lines (A549, NCI-H1299, NCI-H157, GLC-82, and Anip973) were cultured in RPMI-1640 (Gibco, USA) supplemented with 10% fetal bovine serum (FBS). HUVECs were maintained in DMEM (Gibco, USA) supplemented with 10% FBS. All cells were maintained at 37 °C in a humidified incubator under 5% CO_2_ condition. The miR-29c mimic, inhibitor, mimic negative control (NC) and inhibitor NC were purchased from RiboBio (Guangzhou, China). The corresponding sequences were as follows: miR-29c mimic: UAGCACCAUUUGAAAUCGGUUA; miR-29c inhibitor: UAACCGAUUUCAAAUGGUGCUA; mimic NC: UUUGUACUACACAAAAGUACUG; inhibitor NC: UUUGUACUACACAAAAGUACUG. The miRNA transfection was conducted at a concentration of 50 nM using Lipofectamine 2000 (Invitrogen, USA) following manufacturer’s instructions.

### Bioinformatics methods

The potential target genes of miR-29c were predicted using several online programs with databases of different algorithms, including TargetScan (http://www.targetscan.org/), MICRORNA.ORG (http://www.microrna.org/) and MIRDB (http://mirdb.org/).

### Plasmid constructs and luciferase reporter assay

VEGFA expression plasmid pEGFP-N1-VEGFA was generated by cloning open reading frame (ORF) of VEGFA into pEGFP-N1 vector (Generay, China). To construct the VEGFA-3’-UTR-wildtype (WT) plasmid, the 3’-UTR of human VEGFA mRNA including predicted miR-29c target binding sequences was amplified by PCR and cloned into the pGV126 vector (Genechem, China). The VEGFA 3’-UTR-mutant (MUT) plasmid harbored a substation of 12 nucleotides within the binding region of the VEGFA 3’-UTR. In each construct, the 3’-UTR fragment was inserted downstream of the firefly luciferase gene region. A549 cells grown in a 12-well plate were co-transfected with 400 ng of each reporter construct per 20 ng internal control renilla luciferase plasmid (pRL-SV40) and 100 nM of mimic or mimic-NC using Lipofectamie 2000 (Invitrogen, USA). Cells were harvested 48 h after transfection and assayed with the Dual Luciferase Assay kit (Promega, USA) using Synergy H1 hybrid reader (Biotek, USA). Luciferase activity was normalized to the renilla luciferase activity.

### Cell proliferation, migration and invasion assays

The proliferation of the transfected cells was evaluated by Cell Titer 96 AQueous One Solution Cell Proliferation Assay (MTS) (Promega, USA). 2000 transfected cells were seeded in a 96-well plate. After 24 h, 48 h and 72 h, 100 μl 5% MTS was added in a 96-well plate and incubated for 2 h before reading at a wavelength of 490 nm by a microplate reader (Bio-Rad, USA).

The migration and invasion of transfected cells were evaluated by transwell assay. In the migration assay, 5 × 10^4^ (A549) or 10 × 10^4^ (Anip973) cells were cultured in 200 μl serum-free media in the upper chamber of a non-coated transwell 8 μm insert (Costar, USA). For the invasion assay, the upper chamber of the transwell insert was pre-coated with 100 μl of 2% matrigel (BD Biosciences, USA). The lower chamber was loaded with 600 μl RPMI-1640 containing 20% FBS. After 8–12 h, cells that did not migrate or invade were removed with a cotton swab. The migrated cells were fixed in methanol for 10 min and stained with crystal violet for 5 min. Cells in 10 random microscopic fields (×100 magnification) for each insert were counted.

### Preparation of tumor conditioned medium (TCM)

A549 cells transfected with miR-29c mimic or mimic-NC and Anip973 cells transfected with miR-29c inhibitor or inhibitor-NC were cultured in a 6-well plate. 48 h after transfection, the medium was removed. Cells were washed with phosphate-buffered saline (PBS) three times, and then cultured in media free of serum for 24 h. TCM was collected after centrifugation at 12,000 g for 10 min at 4 °C and then stored at −80 °C until used.

### Enzyme-linked immunosorbent assay (ELISA)

The levels of VEGFA in TCM were detected by ELISA kits (R&D Systems, USA) according to the manufacturer’s instruction. TCM was diluted with the sample dilution buffer with a ratio of 1:1. Standard curves were created using purified VEGFA and the CurveExpert 1.3 software program.

### Western blot

Cells were lysed using lysis buffer (1% NP-40 supplemented with a complete protease inhibitor tablet (Sigma, USA)) on ice. 25 μg protein extractions were separated by a 10% SDS-PAGE gel and transferred to polyvinylidene difluoride (PVDF) membranes (Millipore, USA). After blocking with 3% bovine serum albumin (BSA) for 1 h, the membranes were then incubated with primary antibodies overnight at 4 °C: a rabbit polyclonal antibody against VEGFA (1:500, Proteintech, China) and a mouse monoclonal antibody for β-actin (1:5000, Santa Cruz, USA). Afterwards, the membranes were incubated with either anti-rabbit or anti-mouse HRP-conjugated secondary antibody (1:3000 or 1:2000, Sigma–Aldrich, Australia) for 1 h. After incubation with the chemiluminescence substrate, photographs were taken by ImageReader LAS-4000 (Fujifilm, Japan) and analyzed by Image-Pro Plus 6.0 software.

### HUVEC tube formation assay

HUVECs (8 × 10^5^) were suspended in the mixture of TCM (300 ul) and DMEM (300 ul) with 10% FBS and seeded on a 24-well plate coated with matrigel (300 ul/well, BD Biosciences). After 6 h of incubation at 37 °C, tube formation was observed and photographed with a computer-assisted inverted microscope (Nikon). Ten random fields per sample were photographed at × 100 magnification. The number of branch points of the connected tubes was counted and compared between different groups.

### Immunohistochemistry (IHC) analysis

IHC of VEGFA (1:100; Santa Cruz, USA) and CD31 (1:100; ZSGB-BIO, China) was performed in FFPE specimens and visualized by diaminobenzidine (DAB). For VEGFA, the intensity of staining (0, no staining; 1, weakly stained; 2, moderately stained; 3, strongly stained) were assessed by an experienced pathologist and the percentage of positive cells was noted. The microvessel density (MVD) was evaluated by CD31 immunohistochemistry staining. The entire section was scanned at low magnification (100×) to identify four hot spots. MVD was calculated as the average count of microvessel in the four hot spots at high magnification (400×).

### Statistical analysis

Statistical analyses were performed with SPSS version 19.0 or GraphPad Prism version 6.0. Pearson *χ*2 test was used to compare the baseline characteristics. Overall survival (OS) was defined as the time to death or last follow-up from the date of diagnosis. Disease-free survival (DFS) was defined as the time to progression or last follow up or death from the date of diagnosis. Local recurrence disease free survival (LRDFS) was defined as the time to recurrence in the mediastinum, hilum and supraclavicular fossa or last follow-up from the date of diagnosis. Distant metastasis free survival (DMFS) was defined as the time to any recurrence occurring in contralateral lung, brain, bone or other locations or last follow-up from the date of diagnosis. Using the median level of miRNAs as cutoff value, the sample group was classified as high expression group and low expression group. Survival analysis was performed using the Kaplan-Meier method and log-rank test. Univariate and multivariate analyses by use of Cox-proportional hazards model were performed to evaluate potential prognostic factors for OS. For in vitro experiment, difference between groups were analyzed using the Student’s *t*-test (two-tailed). The Spearman’s correlation test was used to examine correlation between miR-29c expression with MVD and VEGFA expression. Data was presented as the mean ± SD. A statistically significant difference was defined as *p* < 0.05.

## Results

### Downregulation of miR-29c confers poor prognosis in IIIA-N2 LAD

To investigate miRNAs’ prognostic role in IIIA-N2 LAD, we first evaluated 1105 known miRNAs’ expression in FFPE specimens using microarray assay from 87 cases with IIIA-N2 LAD who received curative operation as primary treatment in the Cancer Hospital, Chinese Academy of Medical Sciences from 2003 to 2005. Using the median miRNA expression as a cutoff, downregulation of miR-29c was found to be an unfavorable prognostic factor in IIIA-N2 LAD. As shown in Table 1, the clinical characteristics were comparable between the low miR-29c expression group and the high miR-29c expression group.

**Table 1:** Correlation between miR-29c expression and different clinical characteristics

	Patients with low miR-29c expression	Patients with high miR-29c expression	*p*
Age			0.165
< 60 y	27 (61.4%)	20 (46.5%)	
≥ 60 y	17 (38.6%)	23 (53.3%)	
Sex			0.458
Male	27 (61.4%)	23 (53.5%)	
Female	17 (38.6%)	20 (46.5%)	
ECOG performance status			0.740
0	21 (47.7%)	19 (44.2%)	
1	23 (52.3%)	24 (55.8%)	
Weight loss			1.000
≤ 5%	42 (95.5%)	41 (95.3%)	
> 5%	2 (4.5%)	2 (4.7%)	
Smoking status			0.137
Smokers	19 (43.2%)	12 (27.9%)	
Nonsmokers	25 (56.8%)	31 (72.1%)	
Tumor stage			0.324
1	3 (6.8%)	7 (16.3%)	
2	32 (72.7%)	30 (69.8%)	
3	9 (20.5%)	6 (14.0%)	
Postoperative chemotherapy			0.313
Yes	40 (90.9%)	36 (83.7%)	
No	4 (9.1%)	7 (16.3%)	
Postoperative radiotherapy			0.125
Yes	12 (27.3%)	6 (14.0%)	
No	32 (72.7%)	37 (86.0%)	

**Fig. 1:**
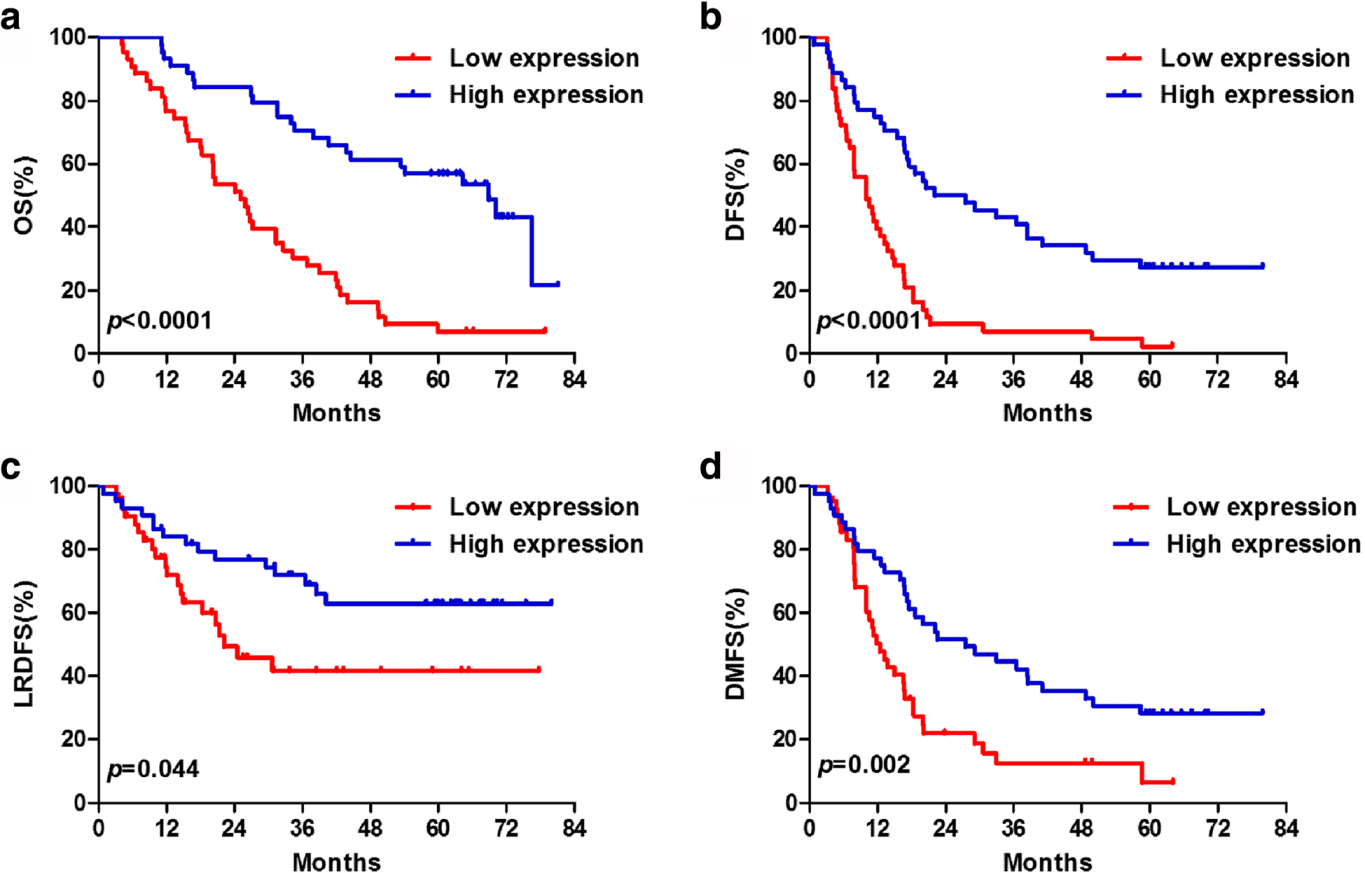
Comparison of survival between the low expression and high expression groups. **a** overall survival (OS), **b** disease-free survival (DFS), **c** local regional disease free survival (LRDFS) and **d** distant metastasis free survival (DMFS)

The median OS and 5-year OS for all patients were 24 months and 26.9%, respectively. Patients in the high expression group achieved significant survival prolongation compared with those in the low expression group (median OS, 68.9 months vs. 25.1 months; 5-year OS, 58.1% vs. 6.8%; *p* < 0.001; Fig. 1a). The median DFS and 5-year DFS for the high expression group (27.5 months and 27.9%) were also superior to those for the low expression group (10.1 months and 2.3%) (*p* < 0.001; Fig. 1b), which was consistent with the OS results. The 5-year LRDFS were 62.4% in the high expression group and 43.5% in the low expression group (*p* = 0.044; Fig. 1c). The 5-year DMFS were 28.9% in the high expression group and 6.0% in the low expression group (*p* = 0.002; Fig. 1d).

The results of the univariate and multivariate analyses of potential prognostic factors for OS are shown in Table 2. Univariate analysis revealed that tumor stage (*p* = 0.005), number of N2 station (*p* = 0.029) and miR-29c expression (*p* < 0.001) were predictors for poor OS. In the multivariate analysis, advanced tumor stage (*p* = 0.033) and downregulation of miR-29c (*p* < 0.001) were significantly correlated with unfavorable OS. These results reveal that reduced miR-29c expression is associated with unfavorable outcome of IIIA-N2 LAD.

**Table 2:** Results of the univariate and multivariate analyses of prognostic factors for OS

	Univariate analysis			Multivariate analysis		
Characteristic	MST (mos)	5-y OS (%)	*p*-value	HR	95% CI	*p*-value
Age			0.394			
< 60 y	31.3	29.8				
≥ 60 y	37.9	35.0				
Sex			0.850			
Male	34.6	30.0				
Female	36.7	35.1				
ECOG performance status			0.987			
0	36.7	30.0				
1	34.0	34.0				
Weight loss			0.593			
≤ 5%	37.9	32.5				
> 5%	31.6	25.0				
Smoking status			0.364			
Smokers	34.6	25.8				
Nonsmokers	36.7	35.7				
Tumor stage			0.005			0.033
1	43.8	50.0		0.456	0.167–1.248	0.126
2	40.7	35.4		0.441	0.237–0.818	0.009
3	15.9	6.7				
Number of N2 station			0.029	0.654	0.387–1.104	0.112
Single	49.3	38.2				
Multiple	26.9	25.0				
Postoperative chemotherapy			0.629			
Yes	34.2	31.5				
No	43.8	36.4				
Postoperative radiotherapy			0.318			
Yes	31.3	22.2				
No	40.7	34.7				
MiR-29c expression			<0.001	3.694	2.106–6.479	<0.001
Low	25.1	6.8				
High	68.9	58.1				

*Abbreviations*: *OS* overall survival, *MST* median survival time, *mos* months, *HR* hazard ratio, *CI* confidence interval

### MiR-29c inhibits cell proliferation, migration and invasion in vitro

**Fig. 2:**
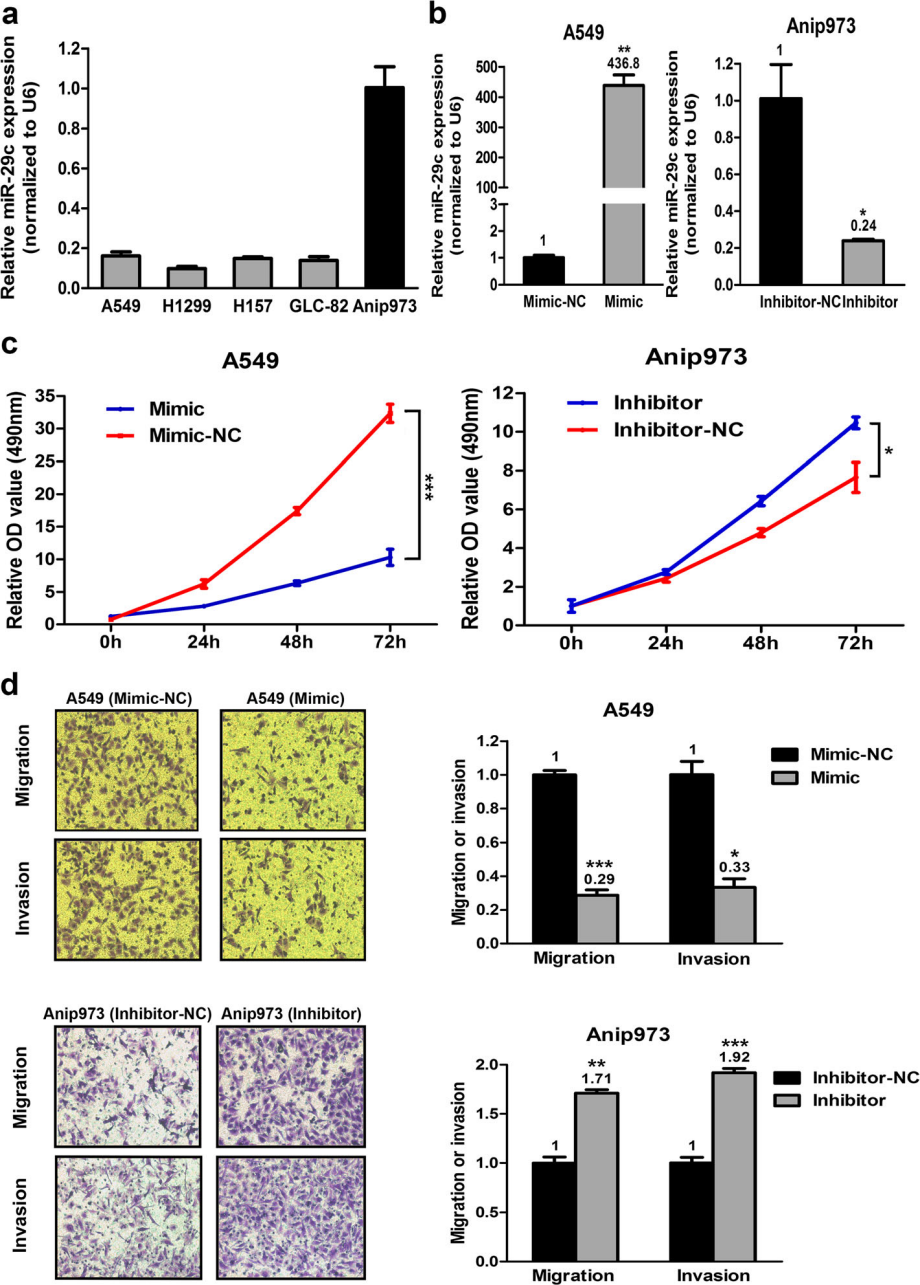
Effect of miR-29c on LAD cell proliferation, migration and invasion in vitro. **a** Expression levels of miR-29c in five LAD cells were analyzed by qRT-PCR. **b** In the A549 cells, miR-29c was overexpressed by the miR-29c mimic. In the Anip973 cells, miR-29c was knocked down by the miR-29c inhibitor. The miR-29c level was evaluated by qRT-PCR. **c** Cell proliferation abilities were determined in A549 cells with miR-29c overexpression and Anip973 cells with miR-29c knockdown compared to those in NC transfected cells. **d** Cell migration and invasion abilities were determined in A549 cells with enforced miR-29c expression and Anip973 cells with reduced miR-29c expression compared to those in NC transfected cells. *p < 0.05, **p < 0.01, and ***p < 0.001

Five LAD cell lines were used to evaluate the expression levels of miR-29c by qRT-PCR. As shown in Fig. 2a, the expression level in the Anip973 cells was significantly higher than that in the other four cell types (A549, NCI-H1299, NCI-H157 and GLC-82) which expressed miR-29c at low levels similarly. We selected A549 cells for miR-29c mimic transfection and Anip973 cells for miR-29c inhibitor transfection, respectively. As shown in Fig. 2b, the miR-29c expression levels were increased by miR-29c mimic in A549 cells and decreased by miR-29c inhibitor in Anip973 cells, respectively. Restoration of miR-29c expression in A549 cells resulted in decreased cell proliferation, whereas inhibition of miR-29c expression in Anip973 cells significantly increased cell proliferation compared with the negative controls (Fig. 2c). The migration and invasion capabilities were increased in Anip973 cells treated with miR-29c inhibitor. Conversely, both capabilities were decreased by miR-29c mimic in 549 cells (Fig. 2d). These results suggest that miR-29c inhibits cell proliferation, migration and invasion in vitro.

### VEGFA is a direct target of miR-29c

**Fig. 3:**
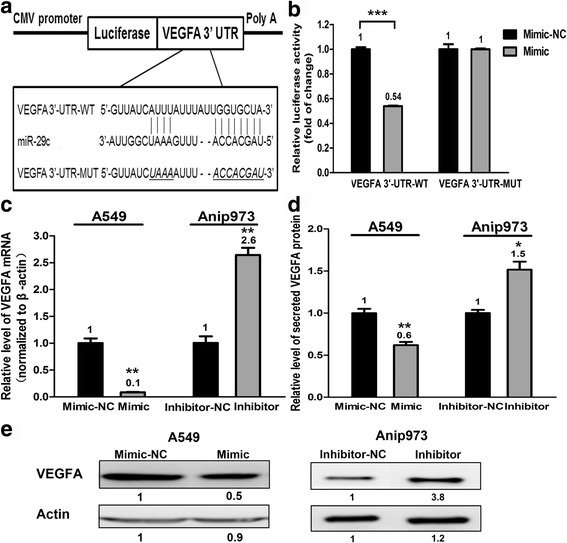
MiR-29c modulates LAD cells through targeting VEGFA. **a** The VEGFA 3’-UTR containing the wildtype or mutant miR-29c binding sequence was inserted into downstream of the luciferase reporter vector. The mutated sequences are in italic. **b** The dual luciferase reporter assay revealed that the luciferase activity controlled by VEGFA 3’-UTR was inhibited by ectopic miR-29c expression in A549 cells. ***p < 0.001. **c** VEGFA mRNA levels determined by qRT-PCR following treatment of A549 cells with miR-29c mimic or Anip973 cells with miR-29c inhibitor. **p < 0.01. **d** ELISA assay of secreted VEGFA protein levels in TCM after treatment of A549 cells with miR-29c mimic or Anip973 cells with miR-29c inhibitor. *p < 0.05, **p < 0.01. **e** Western blot analyses of VEGFA protein levels following treatment of A549 cells with miR-29c mimic or Anip973 cells with miR-29c inhibitor. β-actin was used as control

To further investigate the mechanisms responsible for the tumor-suppressive abilities of miR-29c, we searched TargetScan, MICRORNA.ORG and MIRDB and verified that VEGFA is a potential target of miR-29c. To confirm whether VEGFA was direct target of miR-29c, the wildtype or mutant 3’-UTR of VEGFA was inserted into the downstream of a firefly luciferase reporter gene to create VEGFA 3’-UTR-WT and VEGFA 3’-UTR-MUT (Fig. 3a). Each firefly luciferase vector and renilla luciferase vector (pRL-SV40) was cotransfected with mimic or mimic-NC into A549 cells. The luciferase activity was quantified and compared. As shown in Fig. 3b, the luciferase activity of VEGFA 3’-UTR-WT but not the mutant VEGFA reporter was reduced approximately 50% by miR-29c mimic compared to the mimic-NC, suggesting that miR-29c could bind to the 3’-UTR of VEGFA directly. Then we evaluated whether miR-29c could regulate VEGFA at both mRNA and protein levels (Fig. 3c, d and e). qRT-PCR analysis indicated that overexpression of miR-29c in A549 cells resulted in down-regulation of VEGFA mRNA compared with control. TCM was collected from wells with A549 cells transfected with miR-29c mimic or mimic-NC and Anip973 cells transfected with miR-29c inhibitor or inhibitor-NC. ELISA and western blot analysis revealed that both secreted VEGFA in TCM and intracellular VEGFA protein expression were reduced in miR-29c mimic transfected A549 cells. Furthermore, inhibition of miR-29c in Anip973 cells led to increased expression of VEGFA mRNA and protein. It is concluded that miR-29c represses VEGFA expression at both mRNA and protein levels by directly targeting to its 3’-UTR.

### MiR-29c reduces the capability of tumor cells to promote HUVEC tube formation

**Fig. 4:**
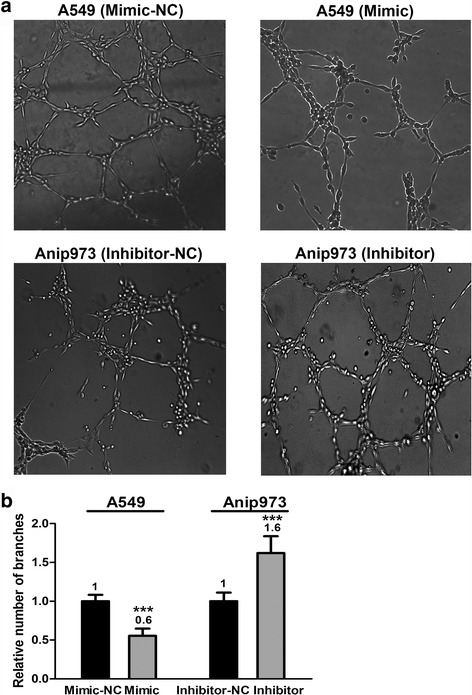
MiR-29c reduces the capability of tumor cells to promote HUVEC tube formation. HUVECs were cultured in TCM derived from A549 cells transfected with miR-29c mimic or mimic-NC and Anip973 cells transfected with miR-29c inhibitor or inhibitor-NC in 24-well matrigel coated plates. **a** and **b** Representative images of tube formation and the relative number of tube branches measured in random 10 photographic fields are presented. ***p < 0.001

To explore whether miR-29c-regulated VEGFA expression is biological significant, HUVEC tube formation assays were carried out using the TCM from LAD cells. HUVECs were suspended in TCM and cultured on the matrigel coated wells to form capillary tubes for 6 h. As shown in Fig. 4, more mature, well-connected capillary tubes were formed in the TCM derived from cells transfected with miR-29c inhibitor. However, less well-organized capillary-like structures were found for HUVECs in the TCM derived from cells transfected with miR-29c mimic. Taken together, the above results suggest that miR-29c inhibits the ability of tumor cells to promote HUVEC tube formation.

### MiR-29c regulates proliferation, migration, invasion and angiogenesis through VEGFA

**Fig. 5:**
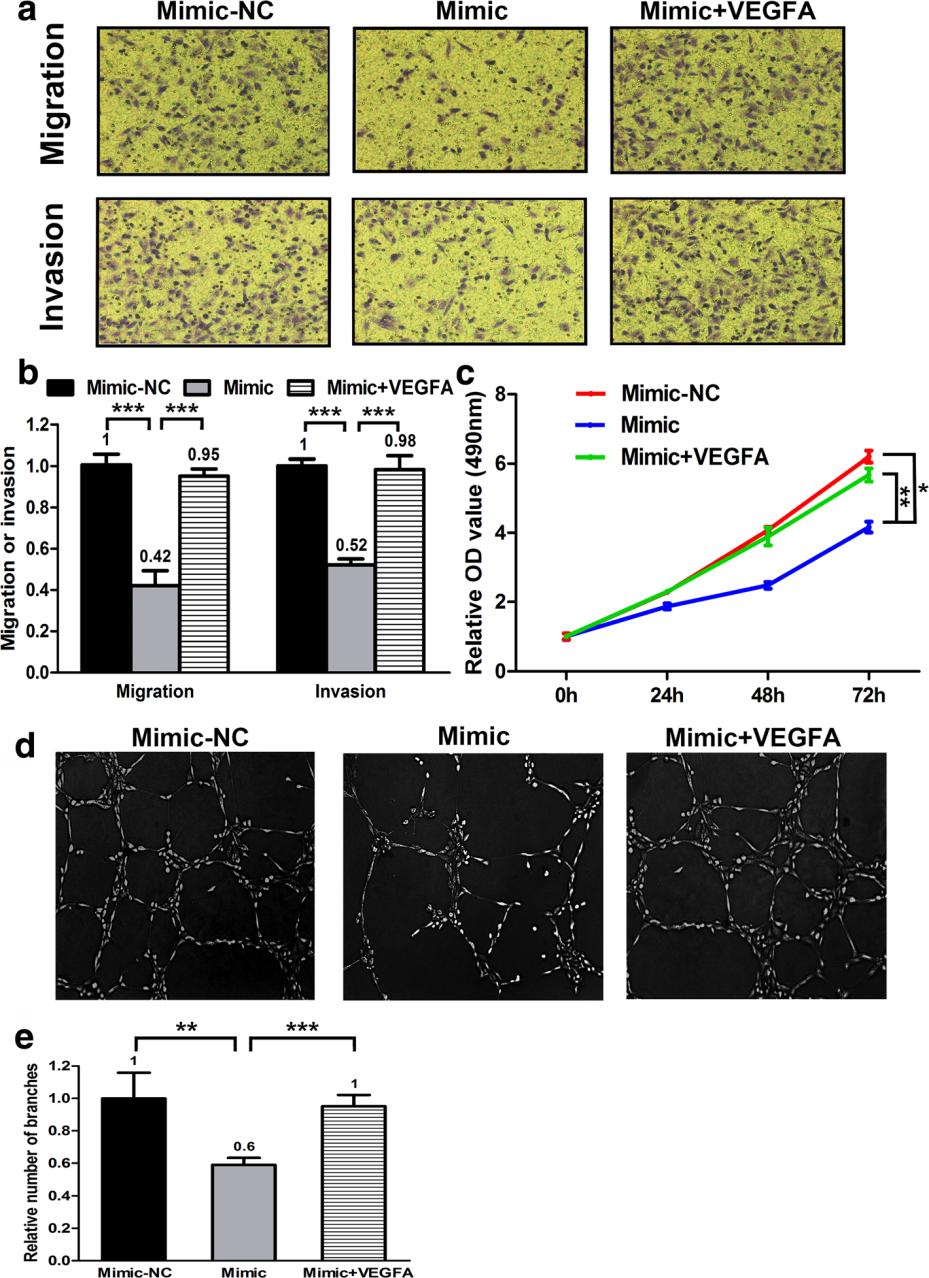
Forced expression of VEGFA abolishes the phenotype created by mimic transfection in A549 cells. **a** and **b** Cell migration and invasion abilities were determined in A549 cells transfected with miR-29c mimic, mimic-NC and miR-29c mimic plus VEGFA expression plasmid. ***p < 0.001. **c** Cell proliferation abilities were determined in A549 cells transfected with miR-29c mimic, mimic-NC and miR-29c mimic plus VEGFA expression plasmid. *p < 0.05, **p < 0.01. **d** and **e** HUVECs were cultured in TCM derived from A549 cells transfected with miR-29c mimic, mimic-NC and miR-29c mimic plus VEGFA expression plasmid. Representative images of tube formation and the relative number of tube branches measured in random 10 photographic fields are presented. **p < 0.01, ***p < 0.001

To investigate the role of VEGFA in proliferation, migration, invasion and angiogenesis of LAD cell, an expression construct carrying VEGFA ORF without 3’-UTR was introduced into A549 cells for overexpression of VEGFA. The compromised cell proliferation, migration, invasion and angiogenesis induced by miR-29c mimic transfection were almost completely reversed by transfection of VEGFA expression plasmid (Fig. 5a-e). These results suggest that miR-29c involves in regulation of cell proliferation, migration, invasion and angiogenesis at least partially depending on regulation of VEGFA.

### Correlation of miR-29c with microvessel density and VEGFA is valid in patients’ samples

**Fig. 6:**
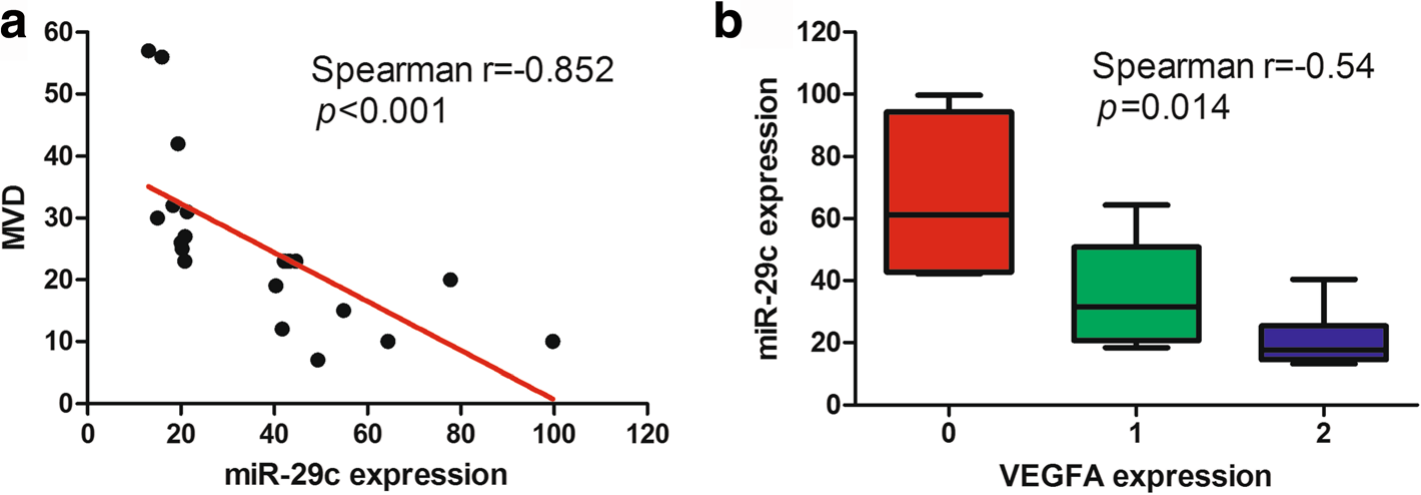
Correlation of miR-29c with MVD and VEGFA expression. **a** Correlation analysis between miR-29c and MVD. *Red line* represents linear regression line. **b** miR-29c expression levels in different VEGFA expression groups

To testify the correlations between miR-29c and MVD, miR-29c and VEGFA expression in tumor tissues, IHC of 20 FFPE specimens for CD31 (MVD) and VEGFA was performed. As shown in Fig. 6, MVD and VEGFA expression correlated significantly with miR-29c expression (*p* < 0.001).

## Discussion

The biological functions of miRNAs in the progression of lung cancer are becoming recognized [18] and there is increasing interest in identifying the key miRNAs involved in aggressive phenotypes of LAD to predict clinical outcome and develop efficient therapeutic strategies. In order to find prognosis related miRNA, we performed miRNA microarray and identified miR-29c expression to be an independent prognostic factor. We found that there was no statistically significant association between miR-29c expression and clinical characteristics. Furthermore, expression of miR-29c was inversely correlated with clinical outcome defined by OS, DFS, LRDFS and DMFS. In addition, the prognostic role of miR-29c expression remained significant after adjusting for clinical parameters in multivariable analysis. To our knowledge, this is the first report to reveal that miR-29c may predict clinical outcome in LAD.

MiR-29c is a member of miR-29 family which also includes miR-29a and miR-29b. MiR-29 family has been observed to be aberrently expressed in different types of cancer and be involved in biological functions including cell proliferation, cell cycle, senescence, differentiation, apoptosis and metastasis [19]. As for lung cancer, growing evidence indicates that miR-29 family functions as tumor suppressor and most studies focus on the biology of miR-29b [8, 20–23]. However, despite the high similarity of miR-29a/b/c mature sequence, studies have revealed that different isoforms of miR-29 family may exert distinct functions [24, 25]. Concerning the tumor suppressive role of miR-29c in lung cancer, it has been demonstrated to revert aberrant methylation by targeting DNA methyltransferases 3A and 3B and inhibit metastasis by targeting integrin beta1 and matrix metalloproteinase 2 [20, 22]. As introduced above, the biological function of miRNA depends on cell types and LAD is a distinct subtype from other histological types. To date, the role of miR-29c in LAD has yet to be thoroughly elucidated.

It is well accepted that miRNAs perform their function by regulating expression of multiple target genes. Bioinformatics analysis revealed VEGFA may be the functional target gene for miR-29c. We also found that miR-29c directly bound with the 3’-UTR of VEGFA and suppressed VEGFA expression. In addition, we showed that VEGFA regulated by miR-29c was biologically active and influenced HUVEC tube formation. Moreover, we observed the suppression of proliferation, migtation/invasion and angiongenesis by enforced miR-29c expression were almost completely rescued by overexpression of VEGFA in A549 cells. The correlation of miR-29c with MVD and VEGFA was further confirmed in patients’ samples. For the first time, these results indicate that miR-29c modulates proliferation, migration/invasion and angiogenesis by targeting VEGFA in LAD. Since the miR-29 family members share the same seed sequence, the target genes of miR-29 family may be the same. MiR-29a and miR-29b were reported to target VEGFA in gastric cancer and breast cancer, respectively [26, 27]. The studies above further supported our observations. However, unlike our study, neither of the previous studies have performed angiogenic experiment in vitro nor in vivo to demonstrate the anti-angiogenic role of miR-29.

Pathological angiogenesis is an established hallmark of cancer which promotes tumor growth and metastasis [28]. As a member of VEGF family, VEGFA is the most potent proangiogenic factor. Clinical observations have confirmed that VEGFA is frequently over-expressed in a wide range of tumors and contributes to increased neovascularization and poor prognosis [29]. Meta-analysis revealed that expression of VEGFA is an unfavorable prognostic factor in NSCLC [30]. Nowadays, VEGFA antagonists such as bevacizumab have been used safely in humans alone or in combination with chemotherapy [31]. Bevacizumab, the monoclonal antibody against VEGFA, has been proposed as first line treatment combined with chemotherapy for advanced or metastatic non-squamous NSCLC. The phase III trial Eastern Cooperative Oncology Group (ECOG) 4599 demonstrated prolongation of median overall survival by only two months for the combination of bevacizumab and paclitaxel/carboplatin followed by bevacizumab maintenance compared to chemotherapy-alone [32]. Although bevacizumab could offer an OS benefit, it should be noted that the benefit is modest. Besides VEGFA, miR-29c may target multiple genes and pathways to inhibit tumor growth and metastasis [20, 22, 33–35], implicating miR-29c as a novel promising therapeutic target to provide clinical benefit. The biological effects of VEGF are mediated via binding to specific tyrosine kinase receptors including VEGFR-1 and VEGFR-2. Based on published data, NSCLC cell lines express VEGFR-2, while VEGFR-1 protein is undetectable [36]. The study suggests that miR-29c might modulate proliferation and migration/invasion of lung adenocarcinoma cells by VEGFR-2 which needs further investigation.

## Conclusion

This study demonstrates that miR-29c plays a tumor-suppressive role in LAD and its function is mainly mediated by its target-VEGFA. These findings provide new insight into the underlying mechanisms of LAD progression and highlight miR-29c as a potential prognostic biomarker and therapeutic target of LAD.

## Abbreviations

3’-UTR: 3’-untranslated regions
ALK: Anaplastic lymphoma kinase
BSA: Bovine serum albumin
DFS: Disease-free survival
DMFS: Distant metastasis free survival
EGFR: Epidermal growth factor receptor
ELISA: Enzyme-linked immunosorbent assay
FBS: Fetal bovine serum
FFPE: Formalin-fixed paraffin-embedded
HUVEC: Human umbilical vein endothelial cell
IHC: Immunohistochemistry
LAD: Lung adenocarcinoma
LRDFS: Local recurrence disease free survival
miRNAs: MicroRNAs
MTS: Cell Titer 96 AQueous One Solution Cell Proliferation Assay
MUT: Mutant
MVD: Microvessel density
NC: Negative control
NSCLC: Non-small-cell lung cancer
ORF: Open reading frame
OS: Overall survival
PBS: Phosphate-buffered saline
PVDF: Polyvinylidene difluoride
qRT-PCR: Quantitative real time polymerase chain reaction
TCM: Tumor conditioned medium
VEGFA: Vascular endothelial growth factor A
WT: Wildtype
